# Aspirin Use and Risk of Age-Related Macular Degeneration: A Meta-Analysis

**DOI:** 10.1371/journal.pone.0058821

**Published:** 2013-03-14

**Authors:** Wei Zhu, Yan Wu, Ding Xu, Yan-Hong Li, Ba Jun, Xiao-Long Zhang, Fang Wang, Jing Yu

**Affiliations:** 1 Department of Ophthalmology, Affiliated Tenth People’s Hospital of Tongji University, Shanghai, China; 2 Department of First Clinical Medical College, Nanjing Medical University, Nanjing, Jiangsu, China; Duke University, United States of America

## Abstract

**Background:**

Age-related macular degeneration (AMD) is the main cause of blindness and the curative options are limited. The objective of this meta-analysis was to determine the association between aspirin use and risk of AMD.

**Methods:**

A comprehensive literature search was performed in PubMed, Embase, Web of Science, and reference lists. A meta-analysis was performed by STATA software.

**Results:**

Ten studies involving 171729 individuals examining the association between aspirin use and risk of AMD were included. Among the included studies, 2 were randomized-controlled trials (RCTs), 4 were case-control studies and 4 were cohort studies. The relative risks (RRs) were pooled using a random-effects model. Relative risks with 95% confidence intervals (CIs) of aspirin use as a risk for AMD. The pooled RR of 10 included studies between the use of aspirin and risk of AMD was 1.09 (95% CI, 0.96–1.24). The same result was detected in early and late stage AMD subgroup analysis. In the subgroup analyses, the pooled RR of RCTs, case-control studies and cohort studies were 0.81 (95% CI, 0.64–1.02), 1.02 (95% CI, 0.92–1.14) and 1.08 (95% CI, 0.91–1.28), respectively.

**Conclusions:**

The use of aspirin was not associated with the risk of AMD.

## Introduction

Age-related macular degeneration (AMD) is a progressing disease [1] and it is regarded as the main cause of blindness of elderly patients in the world [2], [3]. Despite closely attention was paid on AMD and the application of anti- vascular endothelial growth factor drug exists nowdays, its pathogenesis is still not fully understood [4] and the curative options are limited [5]. Therefore the new treatment to AMD was included as an important program goal for vision research. Several large-scale epidemiologic studies were conducted to evaluate the possible risk factors of AMD. The most consistent risk factors were increased age, cigarette smoking and family history of AMD [6]–[8]. Meanwhile, cardiovascular diseases, diabetes, high body mass index, vitamin D status, and hyperlipidemia were reported associated with AMD, but the results had not been in full agreement [9]–[14].

Excepted those risk factors mentioned above, aspirin use was also reported to be related with AMD [15]. As an antiplatelet drug, aspirin was considered to be associated with macular hemorrhage in elderly AMD patients, as reported by Kingham et al [15]. However, compared with the above result form a observational study, a set of multicenter collaborative clinical trials demonstrated that there was no difference of hemorrhage rate between users and nonusers [16]. Considering that cardiovascular diseases were possible risk factors and chronic inflammation was a hypothetical mechanism of AMD, aspirin which could decrease the incidence of cardiovascular events [17] and the relationship between aspirin use and incidence of AMD needs further considerations. Several studies were conducted to evaluate the association between aspirin use and incidence of AMD, while no accordant conclusions were drawn [18], [19]. Aspirin is widely used for cardiovascular and cerebrovascular diseases; however, the indistinct association between aspirin use and AMD puzzled clinical judgments. The purpose of this study was to quantify the effects of aspirin use on AMD incidence by meta-analyzing existing studies, and to explore the association between aspirin use and risk of AMD, and to provide quantitative and high-level evidence.

## Materials and Methods

### 1. Search Strategy and Inclusion Criteria

This meta-analysis was conducted according to the PRISMA guidelines [20]. We searched PubMed, Embase and Web of Science to retrieve related studies published before August, 2012 and Medical Subject Heading (MeSH) keywords “aspirin”, “age-related macular degeneration”, as well as keywords “nonsteroidal anti-inflammatory drugs”, “NSAIDs”, “macular degeneration”, “age-related maculopathy”, “maculopathy”, “retinal degeneration”, “drusen”, “choroidal neovascularisation” and “geographic atrophy” were chosen. The citations of related articles were detected for additional publications. When several reports from the same study were published, only the most recent or informative one was included in this meta-analysis. The language was restricted to only English. Contacting to the corresponding authors of retrieved articles was conducted when additional information was needed.

The articles would be considered eligible if the studies met the inclusion criteria: (1) evaluate the association between aspirin use and risk of AMD; (2) adopt a randomized control trial (RCT) or case-control or cohort study design; (3) provide the odds ratios (ORs) or relative risks (RRs) with confidence intervals (CI), standard errors or sufficient data to calculate them.

### 2. Data Extraction and Assessment of Study Quality

Two reviewers (W. Zhu and Y. Wu) extracted the data from each study independently and checked again after the first extraction. Any disagreements about data extraction were discussed by two reviewers and resolved finally. The data extracted from each study contained: name of first author, study design, study period, country, numbers of subjects (cases, controls, or total), adjustments of the related factors, diagnosis of AMD, exposure definition, outcome detected and OR/RR value with 95% CI.

Considering that both RCTs and non-RCTs were included in this current meta-analysis, the methodological qualities of the included studies were assessed by the Downs and Black quality assessment method [21]. The Downs and Black Scale was eligible for both RCTs and non-RCTs and it contains a list of 27 criteria evaluating the reporting, external validity, internal validity-bias, selection bias and power of included studies. Higher score demonstrated higher quality and generally four ranges were grouped as: 26–28, 20–25, 15–19 and ≤14 [22]. The quality scale was assessed by two reviewers (W. Zhu and Y. Zhu) and disagreements were resolved through discussion with the third reviewer (J. Yu).

### 3. Statistical Methods for the Meta-analysis

Expected heterogenicity of the methodology, data source and so on existed in the included studies. Accordingly, random-effects methods were used to pool the association between aspirin use and risk of AMD for all analyses [23]. The effect was combined under the assumption that ORs were accurate approximations of RRs. When both adjusted and unadjusted data were available, the adjusted data (adjusted ORs or RRs with 95% CI) were used to compare the exposed and unexposed of aspirin use. If only stratified results (e.g., by AMD stages) were provided, fixed-effects method was obtained to summarize the results into a single parameter for each study [24]. Meanwhile, subgroup analyses were carried out by AMD stage, adjustment status, study design, site, smoking status, hypertension and hyperlipidemia status. Statistical heterogeneity across studies was evaluated by both the χ^2^ and *I^2^* tests. If *P*<0.1 and *I^2^*>30%, the interstudy heterogeneity was regarded statistically significant. When the heterogeneity couldn’t be ignored, subgroup analyses and meta-regression would be conducted to explore the source of heterogeneity.

Sensitivity analyses were conducted to detect the robustness of the outcome. After excluding the studies with lower Downs and Blacks Scales, the studies with higher quality were included in the sensitivity analyses. Meanwhile, sensitivity analyses were conducted by changing the random-effects methods to fixed-effects methods. Potential publication bias was assessed via both visually evaluating a funnel plot and the Egger test [25], [26]. All the analyses were conducted using the Stata software package (version 11.0; Stata Corp., College Station, TX).

## Results

### 1. Identification and Selection of Studies

The initial 802 articles (312 from PubMed, 411 from Emabse and 79 from Web of Science) were identified. After 275 duplicates and 504 unrelated articles were excluded, 23 full-text articles were assessed for eligibility. Among the 23 articles, 2 articles from the same trials and 13 articles [14], [16], [18],[19],[27]–[35] which didn’t provide available data were excluded. Besides, 2 articles were included from reviewing the reference lists of the related articles. In final, 10 studies [36]–[45] published from 1996 to 2012 were included in this meta-analysis. Figure S1 provided a flow of search results.

### 2. Study Characteristics and Quality

A total of 171729 individuals were included in this current meta-analysis. The characteristics of these included studies were demonstrated in Table 1. Among the 10 included studies, 2 studies were randomized-controlled trials (RCTs), 4 studies were case-control studies and 4 studies were cohort studies. Geographic distribution of all included studies was 4 in Americas, 5 in European and 1 in Asia. The study periods of all the studies differed and the longest period was more than 10 years [37] while the shortest period was only 1 year [44]. In most studies, late stage were defined as neovascularization or geographic atrophy AMD, while the rest AMD patients without late stage AMD was regarded as early stage AMD.

**Table 1:** Characteristics of Eligible Studies.

Author	Study design	Study period	Country	No. of case/control	Adjustment	Diagnosis of AMD	ExposureDefinition	Outcome detected	RR/OR (95% CI)	Downs and Black Scale					
										Reporting	Externalvalidity	Internalvalidity-bias	Internalvalidity-confounding	Power	Total
Douglas^38^	Case-control	1987–2002	UK	104176	Consultation Rate	By specialists	Ever or never	Any stage	1.00 (0.96–1.04)	6	1	3	1	4	15
Christen^39^	RCT	1996–2006	US	19934/19942	Age and vitamin E and beta carotene treatment assignment	Questionnaires	100 mg of aspirinon alternate days	Current use	0.96 (0.84–1.10)	8	1	4	3	5	21
Rudnicka^40^	Case-control	1996–2002	UK	158	Age, sex, and smokingstatus	Fundus photography	Any dose ofaspirin	Late stage	0.47 (0.20–1.08)	4	2	4	4	0	12
Christen^41^	RCT	1988–1995	US	10617/10599	None	Questionnaires	325 mg everyother day	Any stage	0.77 (0.44–1.32)	7	1	3	4	5	20
de Jong^42^	Case-control	2000–2003	European	4691	None	Fundus photography	Ever or Never	Early and late stage	1.84 (1.39–2.41)	6	0	3	2	4	15
Klein^47^	Cohort	1988–1990∼ 2008–2010	US	693/2513	Age, sex	Fundus photography	twice per more than 3 months	Early and late stage	1.01(0.79–1.23)	6	2	3	4	4	17
Hirvela^43^	Cohort	1995	Finland	195/262	Age	Fundus photography	Ever or never	Any stage	1.04 (0.72–1.51)	5	1	4	3	2	15
Wang^44^	Cohort	1992–1994	UK	1282/2372	Age, sex, smoking andAMD family history	Fundus photography	Current users, past users or ever users	Early and late stage	1.21 (0.98–1.50)	6	2	3	3	3	17
DeAngelis^45^	Case-control	1998–2003	US	73/73	None	Fundus photography	At least 6 months before diagnosis	Any stage	1.37 (0.57–3.32)	7	2	2	3	1	15
Moon^46^	Cohort	2010	Korean	10449	None	Fundus photography	Ever or never	Early stage	1.22 (0.86–1.73)	2	1	4	3	4	13

RR: relative risk; OR: odds ratios; CI: confidence interval; AMD: age-related macular degeneration.

Six studies provided adjusted RR/OR value and the adjusted factors (e.g., age, sex and smoking status) were different in each study. The definitions of aspirin use differed in each studies and the most common was ever use of aspirin. However, 100 mg or 325 mg of aspirin every other day and at least 6 months before diagnosis were also adopted in several studies. To evaluate the methodological qualities of the included studies, the Downs and Black Scale was used in current meta-analysis. The Downs and Black quality assessment scores of the most studies were over 14 (mean: 16.00; standard deviation: 2.83) and 2 studies got less than 14 because of the shortages in data source and methodological designs.

### 3. Aspirin Use and Risk of AMD

Figure S2 showed the effect of aspirin use and risk of AMD. In a random-effects meta-analysis, the use of aspirin was not associated with risk of AMD (RR, 1.09; 95% CI, 0.96–1.24; *I^2^*,67.3%). Table 2 displayed the effects of aspirin use and AMD risk in subgroup analyses by AMD stages, adjustment status, study type, country, smoking status, hypertension and hyperlipidemia status. The results of subgroup analysis of the stages of AMD demonstrated that aspirin use was corrected with neither early stage (RR, 1.02; 95% CI, 0.87–1.20; *P* = 0.872; *I^2^* = 54.7%) nor late stage AMD (RR, 1.11; 95% CI, 0.77–1.60; *P* = 0.587; *I^2^* = 63.3%). In neither adjusted (RR, 0.99; 95% CI, 0.86–1.34; *I^2^*, 29.5%) nor unadjusted (RR, 0.99; 95% CI, 0.87–1.12; *I^2^*, 89.8%) groups, no significant relation between aspirin use and incidence rate of AMD was observed. When subgroup analyses were conducted according to the study types, no associations were detected in RCT group (RR, 0.81; 95% CI, 0.64–1.02; *I^2^*, 0.00%), case-control group (RR, 1.02; 95% CI, 0.92–1.14; *I^2^*, 44.8%) and cohort group (RR, 1.17; 95% CI, 0.96–1.14; *I^2^*, 0.00%).

**Table 2:** Subgroup Analysis of Aspirin Use and AMD risk With Combined RR.

	Subgroups	No. of studies	No. of cases	Summary Effect		Study Heterogeneity	
				RR (95% CI)	*p* Value	*I^2^*, %	*p* Value
AMD stage	Early stage	5	21948	1.02(0.87–1.20)	0.782	54.7	0.065
	Late stage	5	9620	1.11(0.7–1.60)	0.587	63.3	0.028
Adjustment status	Adjusted	7	135293	0.99 (0.86–1.34)	0.853	29.5	0.203
	Unadjusted	9	176036	0.99 (0.87–1.12)	0.847	89.8	<0.001
Study type	RCT	2	45092	0.81 (0.64–1.02)	0.073	0	0.838
	Case-control	4	109171	1.02 (0.92–1.14)	0.666	44.8	0.143
	Cohort	4	17766	1.08 (0.91–1.28)	0.126	0	0.688
Site	Europe	5	113136	1.04 (0.93–1.16)	0.509	45.3	0.12
	Americas	4	48064	0.88 (0.72–1.08)	0.213	0	0.513
	Asia	1	10449	1.22 (0.86–1.73)	0.682	–	–
Smoking cigarettes	Yes	2	52811	0.80 (0.80–1.45)	0.219	0	0.745
	No	2	87297	0.83 (0.64–1.07)	0.149	0	0.507
Alcohol use	Yes	2	94585	0.88 (0.66–1.16)	0.347	22.8	0.255
	No	2	50162	0.83 (0.64–1.07)	0.149	0	0.507
Hypertension	Yes	2	24071	0.63 (0.23–1.74)	0.374	78.4	0.03
	No	2	120641	0.81 (0.63–1.05)	0.109	0	0.33
Hyperlipidemia	Yes	2	17926	0.82 (0.55–1.21)	0.314	0	0.348
	No	2	119178	0.81 (0.63–1.04)	0.096	0	0.561

AMD: age-related macular degeneration; RR: relative risk; CI: confidence interval.

In 2 included studies that provided the data about the hypertension subgroup analysis, the effects of aspirin use on the incidence of AMD in patients with hypertension were 0.99 (95% CI, 0.67–1.46) and 0.35 (95% CI, 0.15–0.83); while the data in the patients without hypertension were 0.73 (95% CI, 0.52–1.01) and 0.95 (95% CI, 0.63–1.44). The pooled result showed that not significant correlations were observed in patients with (RR, 0.63; 95% CI, 0.23–1.74; *P* = 0.374; *I^2^* = 78.4%) or without (RR, 0.81; 95% CI, 0.63–1.05; *P* = 0.109; *I^2^* = 0%) hypertension. However, the results didn’t change in the subgroup analyses by country, smoking status and hyperlipidemia.

A significant heterogeneity was observed when all the 10 studies were included (*I^2^*, 67.3%; *P*<0.001). Conducting the subgroup analysis, the heterogeneity was also significant when early stage AMD (*I^2^* = 54.7%, *P* = 0.065), late stage AMD (*I^2^* = 63.3%, *P* = 0.028), unadjusted subgroup (*I^2^*, 89.8; *P*<0.001), case-control group (*I^2^* = 44.8%, *P* = 0.143), the European site (*I^2^* = 45.3%, *P* = 0.12), hypertension group (*I^2^* = 78.4%, *P* = 0.03) were studied. A sensitivity analysis was conducted after 2 studies which got Downs and Black Scales <15 were excluded and no change of result was observed (RR, 1.02; 95% CI, 0.95–1.09; *I^2^*, 14.8%). There was also no change when the fixed-effects method was obtained with all the 10 studies included (RR, 1.01; 95% CI, 0.97–1.05; *I^2^*, 28.1). No significant publication bias was found in the selected 10 studies (Begg’s funnel plot, symmetrical; Begg’s test, *P* for bias = 0.180; Egger’s test, *P* for bias = 0.906).

## Discussion

The finding of this current meta-analysis including RCTs, case-control and cohort studies, indicated that the use of aspirin was unlikely associated with risk of AMD. Meanwhile, the subgroup analyses by adjustment status, study type, country, smoking cigarettes, alcohol use, hypertension and hyperlipidemia status didn’t provide changed results. The majority studies showed hazard effects in Table 1 while in the subgroup analysis, protect effects were noted even the effect isn’t significant. A possible explanation is that only a relatively small number studies provided data in the subgroup analysis. In all the subgroups, more than half groups contained only two studies, so it should be more cautious to get the conclusions from this subgroup analysis.

Considering the different stages of AMD, neither early stage AMD nor late stage AMD was correlated with the use of aspirin. However this result should be considered cautiously because the diagnosis of AMD was different in each study. Although neither adjusted nor unadjusted group showed a significant association between the use of aspirin and risk of AMD, the heterogeneity in the unadjusted group was quite significant. It could be inferred that the baselines in each study were quite different and the unadjusted factors increased the interstudy heterogeneity. The pooled estimate of the two RCTs showed a moderate protective effect (RR, 0.81), while the results of the case-control and cohort subgroups demonstrated slight harmful effects (RR, 1.02 and 1.17). This finding between the different study designs can be explained by the methodological quality; however, the results of the subgroups analyses were nonsignificant and the number of included studies in the RCTs subgroup relatively few, the results should be interpreted with caution.

Aspirin was an antiplatelet drug widely used and it was reported that aspirin use was a primary prevention of several kinds of cancer [46], [47] and other diseases recently [24], [48]. In the Early Treatment Diabetic Retinopathy Study, possible beneficial effects of aspirin therapy were examined; however, neither beneficial nor harmful effects were indicated [49]. The association between aspirin use and AMD risk was studied several decades before. In 1986, el Baba F and collaborators reported 15 cases of AMD complicated by massive intraocular hemorrhage. Clinicopathologic correlation and histologic findings were presented and the use of aspirin might predispose the patients to serious ocular hemorrhagic complications [50], several following studies demonstrated contradictory results of the association between aspirin use and occurrence of ocular hemorrhage [16], [51], [52] and no definitive conclusion was reached.

The Blue Mountains Eye Study [42] including 3654 participants was conducted to explore the relation between anti-inflammatory medications and AMD. Through baseline examination and 5-year follow-up examination (75% of the surviving participants), anti-inflammatory medications was not associated with neither prevalent nor incident AMD after adjusting for age, sex, smoking and AMD family history. The Age-Related Eye Disease Study (AREDS) [10], [33], a cohort study, investigated the possible risk factors for AMD and nonsignificant association between aspirin use and risk of AMD except central geographic atrophy was detected. A nominally significant (*P*<0.15) age-, gender-, AMD treatment-adjusted association between aspirin intake and central geographic atrophy was detected. However, no detailed ORs with 95% CI was reported in the AREDS and accordingly this study was excluded from this meta-analysis; however, considering that no significant difference between the results of AREDS and this current meta-analysis and it wasn’t a serious limitation. Recently, the European Eye Study [40], a population-based cross-sectional study, demonstrated interesting results. A total of 4691 participants collected by random sampling were included and aspirin intake was ascertained by a structured questionnaire. After adjusting for age, gender, cardiovascular disease, smoking and cholesterol, frequent aspirin use was associated with risk of AMD and the ORs rose with increasing frequency of consumption. However, nonsignificant association was detected between less frequent use of aspirin (less than daily) and risk of both early and late AMD. In total, this interesting finding warrants further evaluation of the associations between aspirin intake and risk of AMD.

There were also two RCT evaluating the association between aspirin use and AMD: the Physician’s Health Study (PHS) [39] and Women’s Health Study (WHS) [37]. The PHS was conducted to evaluate the contribution of low-dose aspirin (325 mg every other day) and beta carotene (50 mg every other day) compared with the placebo [39]. The AMD was defined as a self-reported data and confirmed by medical record evidence. After 5 years of low-dose of aspirin treatment, the results tend to exclude any large beneficial effects. However, among the participants with hypertension, a significant beneficial effects were evaluated (RR, 0.35; 95% CI, 0.15–0.83). In this current meta-analysis, no significant association between aspirin use and risk of AMD was detected in the hypertension subgroup analyses (RR, 0.63; 95% CI, 0.23–1.74). However, considering only two studies were included in this subgroup analyses and the significant heterogeneity existed, the observation should be evaluated by additional study. The WHS included 39876 females and the patients were followed for about 10 years. All the participants were randomly assigned to receive low dose of aspirin (100 mg on alternate days) and the AMD case was defined as a reduction in best-corrected visual acuity to 20/30 or worse based on self-report confirmed by medical record review. After 10 years of follow up, low does of aspirin demonstrated neither large beneficial nor harmful effects on the risk of AMD; however, this modest but potential important effect required continued examination in other population and both genders. In addition, these two RCT were not designed for detecting the assotions between aspirin use and risk of AMD and the potential existing confounding factors and limitations of the study design couldn’t be avoid. All the epidemiological observational studies and clinical trials mentioned above were in Americas and Europe and only 1 (to our best knowledge) retrospective study reported the association between aspirin use and risk of AMD in Asia [44]. Therefore the requirement of additional epidemiological researches and high-quality clinical trials in Asia or other areas were exigent.

The most recent published article that detected the ues of aspirin and risk of AMD with a more than 10 years follow-up demonstrated that 5 years prior to observed incidence was unrelated with the incidence of AMD; while regular use aspirin of 10 years was small but statistically significant with the increased AMD incidence rate. [45]. This interesting result showed that the relationship between aspirin use and early and late stage AMD is different and aspirin use is a potential risk factor of incidence of late stage but not early stage AMD. In the meanwhile, it is the 10 years’ result but not 5 years’ result showed statistical significance and it should be considered that whether the effect of aspirin use on the incidence of AMD was covered up in previous studies and it needs further investigation.

Several proposed mechanisms may be possible pathway through which aspirin work. Aspirin inhibits both cyclooxygenase-1 and cyclooxygenase-2 and cyclooxygenase-1 was inhibited preferentially. The long-term significant inhibition of platelet aggregability would release the progression of atherosclerosis [53] and it was a potential important pathway. Meanwhile, aspirin treatment is associated with decreased endogenous oxidant stress, enhanced resistance to exogenous peroxide and it extended lifespan of Caenorhabditis elegans [54]. In the pathologic processes of AMD, an important progress, lipid peroxidation, would be released by aspirin. Neovascularization of choroid could also be inhibited by NASIDs through novel anti-stress protein HO-1-dependent pathway [55]. The para-inflammation in the aging retina was concerned and the functional impairments occurred with advanced age could be restored through this process; however, the relationship between para-inflammation and aspirin use was not ever researched.

There were several limitations in this meta-analysis should be considered. Firstly, estimation of aspirin use in most studies was through the self-report questionnaires. The existing recall and selection bias would confound the association between aspirin use and risk of AMD. Secondly, the definitions of aspirin use in the included studies were quite heterogeneous; meanwhile, the dosage and duration of aspirin use in each study were various and it would lead to increasing interstudy heterogeneity. Thirdly, the adjusted factors in each study were different and the pooled estimation of all the included study provided less clear results. Fourthly, the exclusion of the non-English publications might produce language bias. After reviewing the English abstracts, non-English articles in this topic definitively were not detected; accordingly, the exclusion of non-English articles produced no noticeable harm. Finally, the study type of included studies was discordant and the heterogeneity was inevitable; and the pooled result of the included studies would incorporate the existing bias and embody new source of bias [56].

Our study found that aspirin use had neither large protective nor harmful effects for the risk of AMD. However, the results should be considered with cautions because of the limitation listed above. Although no association was detected in this meta-analysis, the contribution of aspirin use on the risk of AMD should be examined through long-term aspirin administration. This interesting and potential important issue needs to be confirmed in future research. Additional high-quality epidemiologic studies are also required to further our understanding the association between the dosage, duration and timing of aspirin use with the risk of both early and late AMD.

## Supporting Information

Figure S1 **Flow diagram showing the identification of relevant studies in the meta-analysis.** The initial 802 articles were identified and after 275 duplicates and 504 unrelated articles were excluded, 23 full-text articles were assessed for eligibility. Two articles from the same trials and 13 articles which didn’t provide available data were excluded. Besides, 2 articles were included from reviewing the reference lists of the related articles. In final, 10 articles were included in this meta-analysis.
(TIF)

Figure S2 **Forest plot of risk estimates of the association between aspirin use and risk of AMD.** In a random-effects meta-analysis, the use of aspirin was not associated with risk of AMD (RR, 1.02; 95% CI, 0.93–1.11; *I^2^*, 28.1%). No significant heterogeneity was observed when all the 10 studies were included (*I^2^*, 28.1%; *P* = 0.186).
(TIF)
