# Correction: Aspirin Use and Risk of Age-Related Macular Degeneration: A Meta-Analysis

**DOI:** 10.1371/annotation/bc36f952-ec0c-45d0-bedd-15429017791e

**Published:** 2013-06-19

**Authors:** Wei Zhu, Yan Wu, Ding Xu, Yan-Hong Li, Ba Jun, Xiao-Long Zhang, Fang Wang, Jing Yu

The authors wish to correct some of the information regarding the methodological aspects of the meta-analysis and the details in Table 1:

The inclusion criteria indicated that studies with case-control or cohort study design were included. This is incorrect as studies with a cross-sectional design were also included. Three included studies, titled "Associations between aspirin use and aging macula disorder: the European Eye Study", "Risk factors of age-related maculopathy in a population 70 years of age or older", and "Prevalence and risk factors of early-stage age-related macular degeneration in patients examined at a health promotion center in Korea" were cross-sectional studies. In the cross-sectional studies, the data for the control group were extracted from the "no aspirin user group".

Cross-sectional studies contain more confounding factors than cohort or case-control studies, this may have a bearing on the results of the meta-analysis and should have been noted as a limitation.

The numbering of the references in the Table 1 is incorrect; the numbers of the references in the Table should read 36, 37, 38, 39, 40, 45, 41, 42, 43, 44 and 45.

