# Supplementary figures and images for: Aspirin Use and Risk of Age-Related Macular Degeneration: A Meta-Analysis
Source: PLoS One. 2013 Mar 14;8(3):e58821. doi: 10.1371/journal.pone.0058821 (PMC3597550; doi:10.1371/journal.pone.0058821)

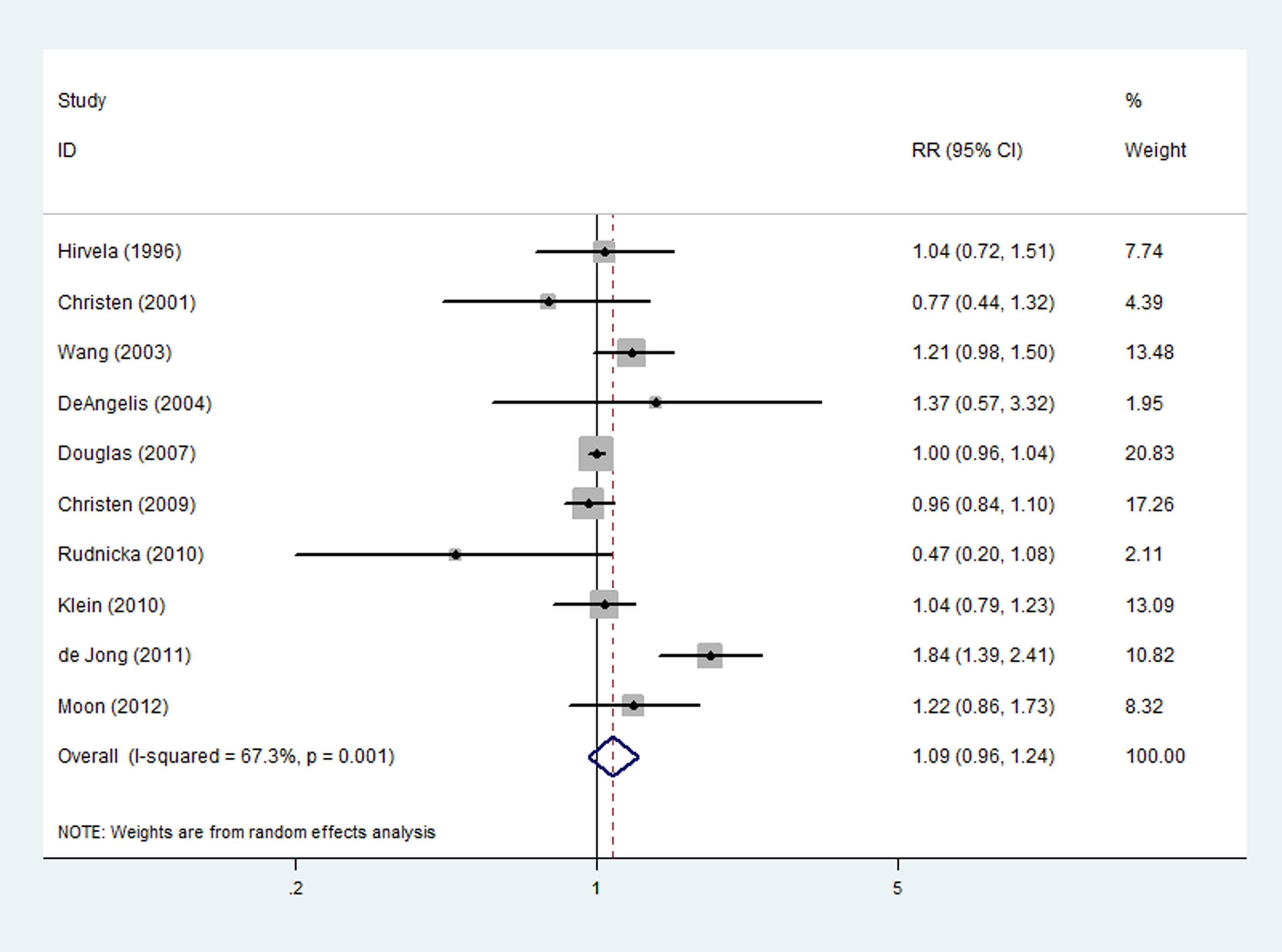

Supplement: Figure S2 — Forest plot of risk estimates of the association between aspirin use and risk of AMD. In a random-effects meta-analysis, the use of aspirin was not associated with risk of AMD (RR, 1.02; 95% CI, 0.93–1.11; I2, 28.1%). No significant heterogeneity was observed when all the 10 studies were included (I2, 28.1%; P = 0.186). (TIF) [file pone.0058821.s002.tif]
